# A descriptive study of prevalence, clinical features and other findings of neuromyelitis optica and neuromyelitis optica spectrum disorder in Khuzestan Province, Iran

**Published:** 2015-10-07

**Authors:** Davood Kashipazha, Seyed Ehsan Mohammadianinejad, Nastaran Majdinasab, Mostafa Azizi, Majid Jafari

**Affiliations:** ^1^ Department of Neurology, Golestan Hospital, Ahvaz Jundishapur University of Medical Sciences, Ahvaz, Iran

**Keywords:** Neuromyelitis Optica, Multiple Sclerosis, Epidemiology, Prevalence, Iran

## Abstract

**Background:** Neuromyelitis optica (NMO) is an uncommon neuro-inflammatory syndrome that has shown to be distinct from multiple sclerosis (MS) and associated with the autoantibody marker NMO-immunoglobulin G (IgG). There are still only a few studies regarding the epidemiology of NMO in Iran. In the present study, we tried to describe the epidemiology of NMO in Khuzestan as one of the densely populated regions in Iran.

**Methods: **A cross-sectional study was performed during the period 2013-2014. Multiple regional sources of data were used including hospital records, details from neurologists and MS society database. The diagnosis of NMO was based on clinical presentation, abnormal findings on neuroimaging and serological tests.

**Results: **A 51 Caucasian patients (36 patients with NMO and 15 with NMO-spectrum disorder) were identiﬁed with a female/male ratio of 7.5:1.0. The crude prevalence of NMO was 1.1/100,000 population. The mean age at onset was 29.2 ± 6.1 years and the mean duration of symptoms was 5.0 ± 0.4 years. The majority of patients (60.8%) were classified as having mild disability (Expanded Disability Status Scale = 0-3.5). Among of 35 patients whose titer of NMO-IgG was measured, 19 (54.2%) were seropositive.

**Conclusion:** Our study suggests that NMO prevalence rate in South West Iran (Khuzestan Province) is much lower than that reported for MS prevalence rate (16.2/100,000) and our patients had a lower age at onset presentation and milder course of the disease than western countries.

## Introduction

Neuromyelitis optica (NMO) or Devic’s disease is a severe inflammatory demyelinating disease of the central nervous system that preferentially targets the spinal cord and optic nerves. For a long time, this disease was regarded as a severe variant of multiple sclerosis (MS), but recent investigations show that it is a distinct disease with humoral pathogenic mechanisms. Devic and his student Gault, in 1894, first reported the clinical characteristics of NMO, optic neuritis (ON), and acute transverse myelitis (TM), based on 16 cases from the literature as well as a fatal case from his own experience.^1^ Since the first description of NMO, it was considered as severe monophasic syndrome characterized by bilateral ON and myelitis occurring simultaneously or in rapid succession, but subsequent studies reported a relapsing course which ultimately resulted in paraplegia and blindness. NMO spreads worldwide and poor prognosis is still a challenge.^2^ In addition to clinical, laboratory, immunological, and pathological characteristics which are mainly used to distinguish NMO from MS, a serum autoantibody termed NMO-immunoglobulin G (IgG), which targets the astrocytic water channel aquaporin-4 (AQP4), has recently been characterized for more differentiation. Therefore, detection of this highly specific marker in the serum of patients with NMO, has helped to define a NMO spectrum of disorders (NMOSD).^3^

Although this disease has been reported in several countries and racial groups, knowledge on the incidence and prevalence of NMO is still limited. The disease is 3-9 times more prevalent in women than it is in men, and the average age of onset ranges from 35 to 45 years among adults. Monophasic form of NMO affects females and males equally, however in the relapsing form, females (ratio 5:1-10:1) are over represented.^4^ Population based studies from Japan,^5^ Cuba,^6^ Denmark,^1^ Mexico,^7^ and the French West Indies^8^ calculated prevalence rates of 0.52-4.40 per 100,000 patient-years and incidence rates of 0.053-0.400 per 100,000 people. The disease is more common in Asian, Indian and black populations, in which NMO forms 15-57% of demyelinating diseases.^4^ It is estimated that NMO prevalence in the United States is approximately 1-2% that of MS, with a female/male ratio of 6.5:1.0 in and a seropositivity of 68.3%.^9^ The disease is mainly sporadic; however, a few studies have reported familial cases.^10^

There’s little epidemiological study about NMO in Iran. Khuzestan is the most densely populated province in southwestern Iran where, to date, no epidemiological data on NMO exist, which emphasizes the importance of epidemiological assessments in this area. The present study aims to description of prevalence, clinical characteristics and other finding of NMO and NMOSD patients in Khuzestan.

## Materials and Methods

The Khuzestan province is situated in the southwest of Iran with a population of over 4.5 million of which 2.28 million are male and 2.24 million are female according to the national census in 2010.^11^ Most of the population is between 15 and 35 years, mainly 20-25 years. Khuzestan is inhabited by a number of ethnic groups: Lors (including Bakhtiari people) Arabs, Persians, Turks, and Kurds. This province is located in a subtropical/tropical area with humid and hot weather in summer and dry-cold weather in winter. The province covers an area of approximately 64,055 km^2^ with most of the area situated over 10 m above sea. Khuzestan borders Iraq and the Persian Gulf and its capital is Ahvaz. The population data used for calculating the prevalence rate was based on the 2010 census, from the Iranian Central Bureau of Statistics. To evaluate the geographical distribution of NMO we divided the province into five regions: North, South, West, East, and Center.

All the patients in our investigation were residents of Khuzestan Province. The study was conducted on patients registered with diagnosis of NMO (during 2006-2014) from the following sources: (1) The records from MS clinic registry of Ahvaz Golestan Hospital, Iran, as the only referral center for NMO and MS patients in the province; (2) the records of patients referred to neurology department of the hospital; and (3) the records from neurologists across the province. We reviewed the medical records of the patients with ON, TM, or NMO, and contacted them to obtain the required details. The patients were also personally re-examined by neurologists to confirm the diagnosis.

We used diagnostic criteria for NMO that required ON and TM plus 2 of the following three supportive elements: (1) Longitudinally extensive TM (LETM) (≥ 3 vertebral segments in length), (2) magnetic resonance imaging (MRI) of the brain with normal findings or with findings not consistent with MS, and (3) NMO-IgG seropositivity.^12^ Requirement of presence of either ON or LETM is challenged in the current definition of NMOSD. It is now evident that brain symptoms are not only frequent during disease course, but may antedate ON or TM for a long time. To diagnosis NMOSDs at least one the following clinical settings were met:

1. Single, recurrent or simultaneous bilateral ON
2. LETM (≥ 3 vertebral segments)
3. Recurrent brainstem symptoms
4. Recurrent hypothalamic symptoms
5. Recurrent cerebral symptoms.

Plus at least 1 of the following:

1. Positive AQP4-IgG serum status
2. Brain MRI lesions typical of NMO.^13^

In our study, only MRIs obtained at the initial clinical event were assessed. Brain MRIs classified as being normal or atypical for MS. Lesions in the spinal cord were characterized as extending over three or more vertebral segments and mainly located in the cervical and thoracic cord with a central gray pattern. NMO-IgG test was done on the serum samples and results were recorded as seropositive or seronegative.^4^ The degree of neurologic impairment in NMO patients was evaluated by Expanded Disability Status Scale (EDSS).^14^ Patients were classified to three groups for EDSS: 0-3.5 (mild disability), 4.0-5.5 (moderate disability), and ≥ 6 (severe disability). Other demographic and clinical parameters which were recorded included: Patient age, gender, ethnicity, age at onset of illness, geographical distribution of illness, disease duration, presentation at onset, course of the disease, family history of NMO, visual evaluation, the use immunosuppressive therapies, type of attacks, history of attacks during pregnancy. Statistical analysis was conducted using SPSS software (version 20, SPSS Inc., Chicago, IL, USA). The frequency or percentage for the nominal variables was calculated. Mean ± standard deviation was calculated for each continuous variable. Exact Poisson confidence intervals (CI) were calculated. The prevalence rate was calculated on November 2013.

## Results

A total of 51 patients were identified with NMO/NMOSD. One of the patients died by time of this report. Patients were exclusively Caucasian and predominantly female, with a female to male ratio of 7.5:1.0. Comparison of the clinical patterns and other finding in our patients based on sex are summarized in table 1. Mean age of patients, mean age at onset and mean EDSS was lower in males than females. The average age at onset of all patients was 29.20 ± 1.55 years (range: 8-58 years), with 10 patients (19%) having an age of onset below 20 years. The mean age of patients was 35.20 ± 1.55 and the duration of the symptoms had a mean of 6.0 ± 1.3 years.

Among the 51 patients, 30 cases (58.8%) were Lors, 13 cases (25.5%) were Arabs, 6 cases (11.8%) were Persians, and 2% were of other ethnic groups (Kurd and Turk). The most cases of NMO disease occurred in the central part (26 patients) as compared to 8 cases in the north, 8 cases in the south, 1 case in the west, and 8 cases in the east of Khuzestan. The crude prevalence of NMO/NMOSD among the Caucasian population living in Khuzestan was 1.1/100,000 (95% CI = 1.04-1.16). Crude prevalence for females and males was 2 (95% CI = 1.92-2.09) and 0.26 (95% CI = 0.23-0.29) per 100,000, respectively. The crude prevalence rate for NMO and NMOSD patients were calculated 0.8 and 0.3 per 100,000 patients (Table 2).

Comparison of the clinical features of the NMO/NMOSD patients are summarized in table 2. Among the 51 patients, 36 cases (70.6%) were identified with NMO and 15 cases (29.4%) with NMOSD. 19 patients (37.3%) presented with ON, 31 (60.8%) with TM and 1 (2%) with simultaneous ON and TM. The disease course was monophasic in 27.5% and a relapsing course was seen in 72.5% of the patients.

Only 2 patients (3.9%) had family history of NMO, who were sisters. Of 4 patients who were attacked during pregnancy, 3 patients had TM and 1 had ON. In our study, 88.2% of patients were on immunosuppressive therapies; of these, 51% used both azathioprine and prednisolone and remaining patients received azathioprine (15.7%), methotrexate (3.9%), cellcept (3.9%), prednisolone (9.8%), and cellcept + prednisolone (3.9%) one of the patients presented NMO with other manifestations such as intractable vomiting. Approximately 12% of our patients received psychiatry therapy.

**Table 1:** Comparison of the clinical patterns and other findings in our patients by sex

Findings	**Men (%)**		**Women (%)**
Number of patients		6 (11.7)	44 (86.3)
NMO		6 (16.6)	30 (83.4)
NMO-Ab positive		1 (16.6)	18 (40.9)
Normal brain MRI		5 (83.3)	31 (70.4)
NMOSD		0	15 (100.0)
Family history of NMO		0	2 (3.9)
EDSS mean		2.25	3.87
Prevalence rate(per 100,000)		0.26	2.00
Age (mean in years)		30.80	35.90
Age at onset (mean in years)		26.00	30.20

NMO: Neuromyelitis opticam; NMOSD: Neuromyelitis optica spectrum disorder; NMO-Ab: NMO antibody; EDSS: Expanded Disability Status Scale; MRI: Magnetic resonance imaging

**Table 2:** Comparison of the patients by type of the disease

Findings	**NMO (%)**	**NMOSD (%)**
Number of patients	36 (70.6)	15 (29.4)
Patients with TM at presentation	19 (52.7)	12 (80.0)
Patients with ON at presentation	16 (44.4)	3 (20.0)
Patients with simultaneous ON + TM	1 (2.9)	0
NMO-Ab positive	15 (42.0)	4 (27.0)
Age (mean in years)	36.3	34.1
Age at onset presentation	31.2	27.2
Female/male ratio	5.0	15.0
Prevalence rate (per 100,000)	0.8	0.3
Mean of EDSS	3.8	3.1

NMO: Neuromyelitis optica; NMOSD: Neuromyelitis optica spectrum disorder; NMO-Ab: NMO antibody; ON: Optic neuritis; TM: Transverse myelitis; EDSS: Expanded Disability Status Scale

**Table 3:** Magnetic resonance imaging (MRI) characteristic of neuromyelitis optica (NMO)/ neuromyelitis optica spectrum disorder (NMOSD)

Findings	**NMO (%)**	**NMOSD (%)**
Brain MRI		
Normal	26 (72.2)	11 (73.3)
Atypical plaques for MS	10 (27.8)	4 (26.7)
Spinal cord MRI		
Normal	1 (3.0)	1 (7.0)
Cervical LETM	32 (82.0)	10 (67.0)
Thoracic LETM	4 (10.0(	2 (13.0)
Cervical plus thoracic LETM	2 (5.0)	2 (13.0)

NMO: Neuromyelitis optica; NMOSD: Neuromyelitis optica spectrum disorder; LETM: Longitudinal extensive transverse myelitis; MRI: Magnetic resonance imaging

**Table 4:** Comparison of the patients by Expanded Disability Status Scale (EDSS)

Findings	**Mild (0-3.5)** **n (%)**	**Moderate (4-5.5)** **n (%)**	**Severe (≥ 6)** **n (%)**
Number of patients	31 (60.8)	9 (17.6)	11 (21.6)
Type of disease			
NMO	21 (67.7)	6 (66.7)	8 (72.8)
NMOSD	10 (32.3)	3 (33.3)	3 (27.2)
NMO-Ab positive	12 (63.0)	3 (16.0)	4 (21.0)
Female/male ratio	26/5 (5.2)	8/1 (8.0)	11

NMO: Neuromyelitis optica; NMOSD: Neuromyelitis optica spectrum disorder; NMO-Ab: NMO antibody

The neuroimaging, neuro-electrophysiological and laboratory findings are listed in table 3. NMO-IgG antibodies were checked for 35 patients. The result was positive in 19 patients and negative in the remaining 16 patients. 15 NMO cases and 4 NMOSD cases were among seropositive patients. Brain MRI findings were characterized as normal findings in 72.5% of patients and atypical plaques for MS was seen in 27.5%. Among the 51 patients underwent spine MRI, 49 (96%) had spinal cord lesions; of these 82.4% had cervical cord lesions; 5.9% had thoracic cord lesions; and 5.9% had lesions spanning the cervical and thoracic cord. Nearly 61% of the all patients had mild disease (EDSS = 0-3.5), 17.5% moderate disease (EDSS = 4.0-5.5), and 21.6% severe disease (EDSS = 6 and above). Because EDSS scores were recorded at the time of clinical visit, the scores for 5 patients with severe disability were obtained when they were experienced an attack (Table 4).

Twenty-five patients underwent visual evoked potential (VEP) test of which 80% had abnormal results. Cerebrospinal fluid oligoclonal bands (OCB) were detected in 4/51 patients (7.8%) and one case was positive for OCB (2%). Three patients showed clinical symptoms and laboratory findings of hypothyroidism. One patient was positive for HLA-B5, which was followed up. Two cases were positive for anti-dsDNA; however in later laboratory tests both had negative results.

## Discussion

In our study, the prevalence of NMO/NMOSD in Khuzestan province was determined to be 1.1/100,000, all of whom were Caucasian. The prevalence of NMO in this region is similar to the prevalence rates in Cuba,^6^ Japan,^5^ South Wales,^15^ and Merseyside (UK),^16^ but lower than Denmark^1^ and Martinique.^8^ A recent report by Etemadifar et al.^17^ in Isfahan, reported a prevalence of 1.9/100,000 for NMO, which is similar to our findings. Our prevalence rate was much lower than that reported for MS in Khuzestan in which a prevalence rate of 16.2/100,000 was observed in 2009^18^ and then prevalence rate of MS in Qom.^19^ The prevalence rates by gender showed a much higher rate in females than in males our results is consistent with the previously reported results of a higher female frequency of NMO.^20^^-^^22^ Female to male rate in our study was 2 times more than the ratio for MS patients living in Khuzestan.^18^

The mean age at onset of illness in this study was 29.2 years (range 8-58) which is very close to those of previous studies in Isfahan and Tehran in which the means of 30 and 27.16 years were suggested.^17^^,^^23^ However, our estimation was lower than the average age of onset in other counties^24^^-^^27^ as well as in the US where a mean of 41.1 years (range 3-81) were reported.^9^

We observed a considerable frequency difference between ethnic groups living in Khuzestan over half of the patients (58.8%) were Lors followed by 25.5% for Arabs. A recent study conducted in Saudi Arabia, found a low frequency prevalence of NMO and a low NMO-IgG seropositivity in a cohort of Saudi patients with Arab ethnic background.^28^ It is believed that Iraq, which has common border with Khuzestan, as well as other Arab countries located in Asia and sub-tropical Africa are classiﬁed as low risk regions for MS.^29^^-^^31^ Sharafaddinzadeh et al.^18^ compared the characteristics of Arab and Persian patients with MS in Khuzestan and observed a much lower prevalence of MS in the Arab ethnic group. The observed ethnic differences between patients with NMO suggest that genetic factors may influence susceptibility to NMO.^5^^-^^8^

In this study, the relapsing form of NMO was more common than the monophasic form (72.5% vs. 27.5%). These findings were similar to prior studies in which 80-90% of the patients followed a relapsing course.^2^^,^^6^^,^^32^ A high proportion of relapsing NMO in Iran was also reported by Sahraian et al.^23^ however Etemadifar et al.^17^ observed monophasic course in 60% of patients. The majority of our patients (60.8%) had EDSS score below 3.5, suggesting a mild course of NMO in our patients similar to the previous reports from Iran^17^^,^^23^ as well a study in Saudi Arabia^27^ but do not support the existing data from around the world which estimated a higher score.^32^^-^^35^ This indicates an aggressive course of NMO in other populations than our population. More patients with MS in Khuzestan had a mild EDSS (< 3.5)^18^ which is in line with NMO patients in our study. In fact, the mean EDSS score in Iranian NMO patients are very close to MS.

In our cohort of NMO patients, there was a family history of NMO in only 2 patients (3.9%) who were two sisters. Similarly, the proportion of patients with positive family history in other studies is low. It is strongly evident that NMO cases appear sporadically; however, recently a few familial NMO cases were reported.^36^ Forty-five patents with NMO received immunosuppressive drugs after diagnosis of NMO of whom 51% used both azathioprine and prednisolone current evidence suggests that the attack prevention is achieved with effective immunosuppressive therapy.^37^

Analysis of the clinical data showed that 4 patients with NMO (7.8%) had an attack while pregnant; one of the patients developed TM 2 months after from delivery and the resting three patients experienced attack during pregnancy. A few reports showed an increase in attack rate in the 1^st^ month after birth.^38^^,^^39^ Several case reports of NMO emerged or became active during pregnancy.^40^^-^^42^ However, there are no studies to indicate the inﬂuence of pregnancy on the long-term course of NMO.

It has been demonstrated that endocrinopathies including hypothyroidism is associated with NMO. Our results showed that 3 patients had symptoms and signs of hypothyroidism and 3 patients were found to be HLA-B5 and anti-dsDNA positive, although this finding was ruled out in serial tests, but indicating a general susceptibility to antibody-mediated autoimmune disease. VEP was performed on 25 patients; of these 20 (80%) had an abnormal VEP response. Previously published data confirms our results by indicating that VEP are frequently altered in NMO.^43^^,^^44^ Existing studies found over 60% of patients with NMO having VEP abnormal results.^28^^,^^43^^,^^44^ Although VEP is not necessary in the diagnosis of NMO, VEP can be helpful and are used commonly in patients with demyelinating disease.^46^ Among 35 patients who took NMO-IgG test, 19 (54.3%) were NMO-IgG seropositive. This rate was close to the range reported from other studies in Caucasian populations suggesting a seropositivity of over seventy percentage.^9^^,^^15^^,^^16^ Other studies from several Asian countries showed a seroprevalence of 27-39%.^45^^-^^49^ Recent studies of seroprevalence of NMO-IgG antibody in NMO from Iran reported proportions of 30-66%.^23^^,^^50^^,^^51^ Which are relatively similar to our estimation. Harirchian et al.,^51^ using cell-based immunofluorescence assay to measure NMO-IgG, demonstrated that NMO antibody is highly specific for NMO but it is not highly sensitive for diagnosing NMO patients. The variable sensitivity across studies can be due to a number of factors including ethnic background, characteristics of patients and more important method of measurements.^52^ The limitations of our study were that the exact population of Lors and Arabs was not available therefore we could not study the relationship between the prevalence of the disease and ethnicity, also some patients may refer to larger city for treatment so we do not have the exact number of patients.

## Conclusion

Our results are in line with other data from western counties in terms of prevalence, NMO features, MRI ﬁndings, laboratory results, and female preponderance. However, the lower age of onset and milder course of the disease are exceptions in our patients. Overall, patients in Khuzestan appear to have similar characteristics to Caucasians living in Asia and western countries.

## Notes:

**How to cite this article: **Kashipazha D, Mohammadianinejad SE, Majdinasab N, Azizi M, Jafari M. A descriptive study of prevalence, clinical features and other findings of neuromyelitis optica and neuromyelitis optica spectrum disorder in Khuzestan Province, Iran. Iran J Neurol 2015; 14(4): 204-10.
